# Cancer incidence in relatives of British Fanconi Anaemia patients

**DOI:** 10.1186/1471-2407-8-257

**Published:** 2008-09-11

**Authors:** Marc Tischkowitz, Douglas F Easton, Jan Ball, Shirley V Hodgson, Christopher G Mathew

**Affiliations:** 1 Cancer Genetics Program, Departments of Human Genetics and Oncology, Sir M.B. Davis Jewish General Hospital, McGill University, Montreal, Quebec, Canada; 2 Department of Medicine, McGill University, Montreal, Quebec, Canada; 3 Cancer Research UK Genetic Epidemiology Research Group, Strangeways Research Laboratory, Cambridge, UK; 4 Division of Genetics and Molecular Medicine, King's College London School of Medicine, Guy's Hospital, London, UK; 5 Department of Medical Genetics, St Georges Hospital University of London, London, UK

## Abstract

**Background:**

Fanconi anemia (FA) is an autosomal recessive DNA repair disorder with affected individuals having a high risk of developing acute myeloid leukaemia and certain solid tumours. Thirteen complementation groups have been identified and the genes for all of these are known (*FANCA, B, C, D1/BRCA2, D2, E, F, G, I, J/BRIP1, L, M *and *N/PALB2*). Previous studies of cancer incidence in relatives of Fanconi anemia cases have produced conflicting results. A study of British FA families was therefore carried out to investigate this question, since increases in cancer risk in FA heterozygotes would have implications for counselling FA family members, and possibly also for the implementation of preventative screening measures in FA heterozygotes.

**Methods:**

Thirty-six families took part and data was collected on 575 individuals (276 males, 299 females), representing 18,136 person years. In this cohort, 25 males and 30 females were reported with cancer under the age of 85 years, and 36 cancers (65%) could be confirmed from death certificates, cancer registries or clinical records.

**Results:**

A total of 55 cancers were reported in the FA families compared to an estimated incidence of 56.95 in a comparable general population cohort, and the relative risk of cancer was 0.97 (95% C.I. = 0.71–1.23, p = 0.62) for FA family members. Analysis of relative risk for individual cancer types in each carrier probability group did not reveal any significant differences with the possible exception of prostate cancer (RR = 3.089 (95% C.I. = 1.09 – 8.78; Χ^2 ^= 4.767, p = 0.029).

**Conclusion:**

This study has not shown a significant difference in overall cancer risk in FA families.

## Background

It has long been hypothesised that heterozygotes for autosomal recessive DNA repair disorders may have reduced efficiency of their DNA repair systems which could cause an increased risk of cancer [1]. This hypothesis has been upheld by molecular and epidemiological studies of cancer incidence in Ataxia Telangiectasia which have confirmed a two-fold increased risk of breast cancer in female *ATM *heterozygotes [2,3]. Fanconi anemia (FA) is an autosomal recessive bone marrow failure syndrome due to mutations in at least 13 different genes (*FANCA, B, C, D1/BRCA2, D2, E, F, G, I, J/BRIP1, L, M *and N/PALB2) [4,5] and the incidences of acute myeloid leukaemia and certain solid tumours are all greatly increased in homozygotes [6]. Epidemiological studies of cancer risk in FA heterozygotes are conflicting: the initial study by Swift *et al. *analysed 102 deaths in the relatives of eight FA families and found a higher rate of leukaemia and gastric, colorectal and tongue cancer [7]. However, none of these was statistically significant and when the study was expanded to 25 families no overall or specific excess of cancers could be demonstrated, and indeed there were fewer leukaemias than expected [8]. A separate study of 125 relatives in nine FA families also failed to show a difference [9]. While molecular studies investigating cancer risk in heterozygotes have mostly been inconclusive (reviewed in [10]), a recent molecular study of 944 relatives of FA probands did suggest an increased risk of breast cancer in *FANCC *mutation carriers [11].

In view of these conflicting data we carried out a study of British FA families to investigate this question further, since increases in cancer risk in FA heterozygotes would have implications for counselling FA family members, and possibly also for the implementation of preventative screening measures in FA carriers.

## Methods

### Data collection

Families with individuals affected with FA were traced by contacting haematologists with an interest in FA and clinical geneticists throughout the UK as well as using the resources of the FA patient support group. The study had Multi Centre Research Ethics Committee approval (MREC 00/1/66). Eligible families were contacted through their hospital specialist or general practitioner and were sent an information sheet and consent form. If they agreed to take part in the study, individuals from these families were asked to fill out a detailed questionnaire about their health and that of their relatives, with particular reference to any malignancies. Where possible, cancer diagnoses were confirmed by obtaining consent from the affected person to contact their clinician, or use of regional cancer registries, or obtaining the death certificate. Cancers were then classified according to the ICD10 criteria [12].

Ninety-six families were identified. Of these, 8 were not suitable (mainly foreign nationals having treatment but not living in the UK), 8 were unwilling to participate, and 14 did not have adequate contact details. Of the remaining individuals from 66 families, 21 did not reply to the invitation to take part in the study and 9 did not return the completed questionnaire. Data was obtained on 695 individuals from 26 "full" families and 10 "half" families, where no data was available on one side of the family because one of the parents was adopted (2 families), not in contact (3 families) or unwilling to participate (5 families). Three families were consanguineous; in two of the families the parents were first cousins and in the third they were second cousins.

### Statistical Analysis

The principal aim of this study was to estimate the risks of cancer in FA gene mutation carriers. A cohort of the following individuals was constructed: (a) FA family members with a carrier probability of 0.25 or greater (up to and including great-grandparents, great uncles and aunts and cousins) who were affected with cancer under the age of 85 years, (b) FA members unaffected with cancer, with a carrier probability of 0.25 or greater.

In order to compute incidence rates for individuals not affected with cancer, follow-up was deemed to commence on their date of birth or 1^st ^January 1960, whichever was the later, and to cease on the date of their first cancer, their date of death or loss to follow-up, their 85^th ^birthday, or 31^st ^December 2001, whichever occurred first. Follow-up before 1960 was ignored to minimise errors in classification of tumours and because reliable population specific incidence rates were available for almost all centres from that date, but often not before. FA homozygotes were also excluded from the analysis.

After removing those cases with no follow-up in the relevant period, the final cohort comprised 575 individuals (276 males, 299 females), representing 18,136 person years. In this cohort, 25 males and 30 females were diagnosed with cancer under the age of 85. Thirty-six (65%) of the total of 55 cancers analysed were confirmed; 20 (36%) from cancer registry information, 9 (16%) from death certificates and 7 (13%) from clinical records. In two out of the 36 cases, the confirmed cancer diagnosis differed from that reported by the relatives (a case of renal cancer which had been reported as liver cancer and a case of colorectal cancer which had been reported as stomach cancer) which implied an error rate of cancer type misreporting by relatives of at least 5.5%.

Expected numbers of cancers were computed in the usual manner by multiplying person-years at risk by the appropriate age, sex, period, site, and population-specific incidence rates, using the programme Person Years [13]. The relevant rates were obtained from combined data from Cancer Registries in the UK. A separate relative risk was estimated for each ten-year age-group 20–29, 30–39, 40–49, 50–59, 60–69, 70–79, relative to population incidence rates. (For this purpose population incidence rates averaged over all centres were used). These estimates were then used to derive the cumulative risk estimates, using previously described methods [14]. Relative risks were also calculated for all cancers in each carrier probability group, and an overall relative risk for carriers was calculated using the EM algorithm [15] as previously described [2].

## Results

The observed versus expected incidence, p-value (Poisson 1-sided) and 95% confidence intervals of the most frequent cancers in the relatives of FA patients are given in Table 1. Head and neck cancer and leukaemias are also listed, as these are common in FA homozygotes. There were no observed cases of other cancers that commonly occur in FA homozygotes (oesophageal, vulval, anal), and other cancers had an observed incidence of 2 cases or less. The results show no significantly different relative risks for any of the cancers and, given the small numbers studied, there is a wide range for many of the associated confidence intervals. The overall observed number of cancers is very similar to the expected number, with a relative risk of 0.97 (0.71–1.23) for FA family members.

**Table 1:** Relative risks, 95% confidence intervals and associated p-values for the most common cancers observed in the study and for those cancers commonly found in FA homozygotes.

**Cancer**	**Observed**	**Expected**	**O/E**	**95% C.I.**	**p-value**
**Breast**	7	8.97	0.78	0.31–1.59	0.795
**Lung**	9	9.3	0.97	0.44–1.84	0.583
**Colorectal**	6	7.1	0.85	0.51–3.04	0.264
**Prostate**	5	3	1.66	0.54–3.89	0.185
**Stomach**	3	2.98	1.01	0.21–2.94	0.573
**Endometrial**	3	1.41	2.13	0.44–6.2	0.168
**Leukaemia**	2	1.29	1.55	0.19–5.6	0.370
**Head and Neck**	3	1.59	1.89	0.12–15.9	0.214
					
**ALL CANCERS**	**55**	**56.95**	**0.97**	**0.71–1.23**	**0.62**

O/E = observed versus expected, C.I. = confidence interval

The relative risk of all cancer in relation to carrier probability is given in Table 2. There were no significant differences in relative risk across the groups, and the rates of all cancers were similar in each group, suggesting that there is little bias in the data. As expected, most of the cancers occurred in the 0.25 carrier probability group, which mainly consisted of great-grandparents and great uncles and aunts. The overall relative risk of all cancers in FA gene carriers was calculated to be 0.946 (95% confidence limits 0.61 – 1.46; Χ^2 ^= 0.0279, p = 0.867).

**Table 2:** Relative risk of cancer in relation to carrier probability

	**Male**				**Female**			
	
**Carrier Probability**	**No. in Group**	**Obs**	**Exp**	**O/E**	**No. in Group**	**Obs**	**Exp**	**O/E**
**1**	33	2	1.86	**1.07**	31	1	1.29	**0.78**
**0.66**	13	0	0.05	**0**	18	0	0.90	**0**
0.5*	109	7	10.04	**0.70**	123	12	12.36	**1.03**
**0.25**	121	16	14.97	**1.07**	127	17	16.27	**1.04**

*This group included 2 nephews and 2 nieces (carrier probability = 0.33), none of whom had cancer.

Obs = number of cancers observed, Exp = number of cancers expected, O/E = observed cases divided by expected cases.

Analysis of relative risk for individual cancer types in each carrier probability group did not reveal any significant differences apart from an increased incidence of prostate cancer in obligate male carriers; 2 cases were observed in 33 individuals when the expected incidence was 0.17 (O/E = 11.5, 95% C.I. = 1.7 – 78.9, p = 0.013). When this was combined with prostate case incidences in other carrier groups there were a total of five cases and the overall relative risk of prostate cancer in carriers was calculated to be 3.089 (95% confidence limits 1.09 – 8.78; Χ^2 ^= 4.767, p = 0.029).

## Discussion

This is the first study of cancer predisposition in UK FA families and our data suggest that there is no overall increased cancer risk in these families. Sixty-five percent of the reported cancers could be confirmed independently, and the cancer-type misreporting rate by relatives was 5.5%. It was not known how many family cancers had been missed by not being reported by relatives, and this could be a potential source of under-ascertainment. Another source of under-ascertainment could result from a situation where a family could not be contacted because one or both of the parents had died from cancer, resulting in a family with a potentially higher cancer incidence not being included in the study. Conversely, over-ascertainment could result from selection bias where relatives who perceived an increased number of cancer cases in their families were more willing to take part. The fact that rates of all cancers are similar in each carrier probability group suggests that the overall level of bias is small.

Three cancers occurred in 64 obligate carriers: a case of follicular thyroid cancer in a female (confirmed by her clinician, diagnosed age 52) and two cases of prostate cancer in males (one confirmed, diagnosed age 71, one unconfirmed, diagnosed age 69). If these observations were extrapolated to calculate relative risk, it would result in significantly increased risks for both cancers in FA mutation carriers. It is quite possible that these are chance occurrences, particularly in the case of the thyroid cancer. The increased incidence of prostate cancer is interesting in view of involvement of *BRCA2/FANCD1 *in the FA gene pathway; male *BRCA2 *carriers have an increased risk of prostate cancer [14] so there is a theoretical molecular link. In the study by Berwick *et al. *[11], there was an increased incidence of prostate cancer in a group that included *BRCA2 *carriers and others of unknown complementation status. Taken together, these two studies suggest that there could be a modest increased risk in prostate cancer in some FA heterozygotes and which merits further investigation.

Our study had a number of limitations that are important to the interpretation of our findings. Complementation data was not available on the majority of the FA families in the study so analysis according to FA gene groups could not be carried out. Given the manner in which these families were ascertained and the ethnic heterogeneity of the UK population, it is likely that the complementation group frequencies would be similar to those previously determined, and that the majority (around two-thirds) of affected families would have *FANCA *mutations. Families where FA is due to biallelic *BRCA2 *mutations will clearly have an increased cancer risk, but these cases are rare (<2% of all FA) and none of the families in this study had a pedigree typical of autosomal dominant breast/ovarian cancer predisposition. It has recently been established that mutations in two other FA genes may confer an increased risk of breast cancer; the increased risk for *FANCJ/BRIP1 *is around 2-fold [16], and that for *FANCN/PALB2 *is probably 2–4 fold [17,18], but may be higher for certain mutations [19]. Like *FANCD1/BRCA2*, both these complementation groups are rare (<1% of FA cases) and interestingly all three of these FA genes act downstream of FANCD2 [20]. In their recently published study Berwick *et al*. found a possible increased incidence of breast cancer in carrier females, with the highest risk in *FANCC *mutation carriers (SIR, 2.4; 95% CI, 1.1–5.2) [1]. No overall increased risk of breast cancer was seen in our study but the number of families with *FANCC *mutations would have been small as this group accounts for less than 10% of non-Ashkenazi Jewish FA cases. A previous study of familial breast cancer cases did not identify mutations in *FANCA, C, D2, E, F, G *[21], so at present it remains unclear whether *FANCC*, which acts upstream of *FANCD2*, is a breast cancer predisposing gene.

## Conclusion

This study did not find any significant difference in cancer incidence in FA families compared to population estimates, with the possible exception of prostate cancer. It therefore seems that only in those rare families with FA cases due to *BRCA2 *or *FANCJ/FANCN *mutations is cancer risk in heterozygotes a salient factor.

## Competing interests

The authors declare that they have no competing interests.

## Authors' contributions

MT coordinated the study, collected and data and wrote the manuscript, DE undertook the statistical analysis, JB assisted with data collection and interpretation, SVH and CGM conceptualised and designed the study, and provided critical revisions to the manuscript. All authors read and approved the final manuscript.

## Pre-publication history

The pre-publication history for this paper can be accessed here:


